# Follicular CD4 T Cells Tutor CD8 Early Memory Precursors: An Initiatory Journey to the Frontier of B Cell Territory

**DOI:** 10.1016/j.isci.2019.09.012

**Published:** 2019-09-14

**Authors:** Marie-Ghislaine de Goër de Herve, Maïsa Abdoh, Salma Jaafoura, Deniz Durali, Yassine Taoufik

**Affiliations:** 1 INSERM 1184, IMVA, Faculté de Médecine Paris-Sud, 63 rue Gabriel Péri, 94275 Le Kremlin-Bicêtre, France; 2 Immunology Research Laboratory, Department of Medical Microbiology, School of Medicine, Istanbul Medipol University, Istanbul, Turkey; 3 Department of Hematology, Bicêtre Hospital, 94276 Le Kremlin-Bicêtre, France

**Keywords:** Immunology, Immune Response, Specialized Functions of Cells

## Abstract

The early events of CD8 memory generation remain largely unknown. Here we report that as early as 2 days after antigen priming, very early memory precursors can be identified by their expression of the chemokine receptor CXCR5. These early precursors, which have an effector phenotype, expand and temporarily migrate to the T-B cell zone junction where they interact with follicular CD4^+^ T cells (Tfh). Remarkably, this interaction with Tfh, hitherto considered as exclusive B cell helpers, is required for CD8 memory precursors to become highly competent memory cells. CD40 and interleukin-21 signaling are involved in the help provided to CXCR5^+^CD8 memory precursors. This study thus unveils critical early steps in the generation of CD8 memory, identifies CXCR5 as the earliest known marker of CD8 memory precursors, suggests a major helper role for Tfh, and points to possible coordination between the pathways of CD8 and B cell memory generation at the T-B-cell zone junction.

## Highlights

• Very early CD8 memory precursors express the chemokine receptor CXCR5
• They migrate to the T-B junction zone where they interact with Tfh
• This interaction with Tfh is required to become highly functional memory cells
• Tfh helping signals involve CD40-CD40 ligand interaction and IL-21 signaling

## Graphical Abstract

**Figure:**
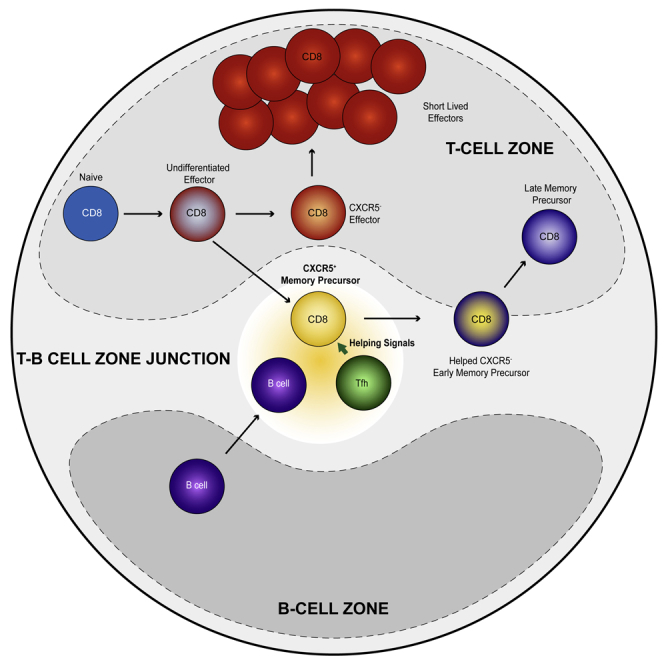

## Introduction

Following antigen activation, a naive CD8 cell subset undergoes strong clonal expansion, generating a heterogeneous population of activated cells that is dominated, at the peak of expansion, by short-lived CD8 effectors (Kaech and Cui, 2012). This expansion is followed by a phase of drastic contraction through massive apoptosis. A few cells survive this contraction phase and eventually become highly competent memory cells (Kaech and Cui, 2012). Precisely when and how these memory precursors are generated is largely unknown, however, and so are the subsequent steps of their maturation into fully functional memory cells. Help signals from CD4^+^ T cells are clearly required throughout the memory precursor (MPEC) maturation process (Laidlaw et al., 2016). However, CD4^+^ T cells form a heterogeneous group that includes several subsets with different functional properties. FoxP3^+^ regulatory CD4^+^ T cells have been shown to favor memory precursor maturation by limiting exposure to interleukin (IL)-2 and by providing inhibitory signals (de Goër de Herve et al., 2012, Laidlaw et al., 2015), but this is probably only one facet of the complex and multifaceted help provided by CD4^+^ T cells to memory precursors (Borst et al., 2018, Laidlaw et al., 2016). Here, we show that, as early as 2 days after antigen priming, very early memory precursors can be identified by their expression of the chemokine receptor CXCR5. These CXCR5^+^ precursors migrate to the junction between T and B cell zones, where they receive critical help from follicular CD4^+^ T cells (Tfh), a specialized CD4^+^ T cell subset hitherto considered as exclusive B cell helpers (Crotty, 2011, Vinuesa et al., 2016). Tfh enable these CXCR5^+^ precursors to become highly competent memory cells.

## Results

### A CXCR5^+^ CD8^+^ Effector Subset Emerges Early after Antigen Priming and Gives Rise to Highly Functional Memory Cells

**Figure 1:**
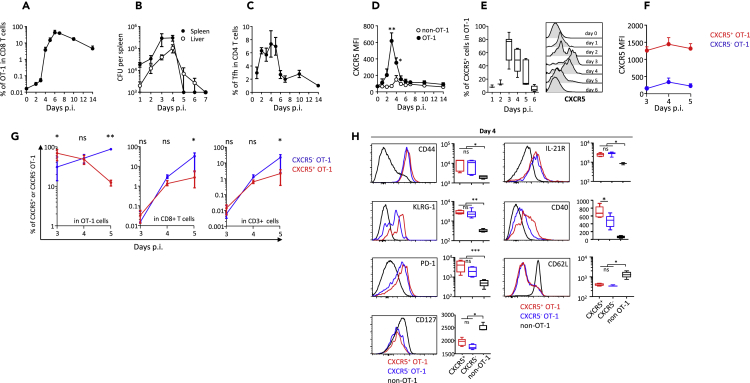
A population of CD8 Primary Effectors Expressing the Chemokine Receptor CXCR5 Is Generated Shortly after rLm-OVA Infection Naive wild-type mice received 10^4^ CD45.1^+^ OT-1 cells and were infected 2 days later with 2 × 10^4^ colony-forming unit of rLm-OVA. (A–C) The frequency of OT-1 cells among CD8^+^ T cells in the spleen (A), the rLm-OVA burden in the spleen and the liver (B), and the frequency of Tfh among CD4^+^ T cells in the spleen (C) at various time points after infection. The data are from three to five independent experiments with at least three mice per time point. (D) The intensity of CXCR5 expression by OT-1 and non-OT-1 CD8^+^ T cells, expressed as mean fluorescence intensity (MFI). Statistical significance of differences between OT-1 and non-OT-1 cells is indicated (Wilcoxon test). (E) The percentage of CXCR5^+^ cells among OT-1 cells. Representative plots are also shown. (F) The intensity of CXCR5 expression (as corrected geometric MFI, i.e., [MFI^OT-1^ – MFI^non-OT-1^]) by CXCR5^+^ and CXCR5^-^ OT-1 cells. (G) The percentages of CXCR5^+^ cells (red) and CXCR5^-^ cells (blue) among OT-1 cells, total CD8^+^ T cells, and total CD3^+^ T cells. Statistical significance is indicated (Wilcoxon test). (H) The geometric MFI of CD44, KLRG-1, PD-1, CD127, IL-21R, CD40, and CD62L expression by CXCR5^+^ OT-1 (red), CXCR5^-^ OT-1 (blue), and non-OT-1 CD8^+^ T cells (black) on day 4 after infection. Representative plots are also shown. The data are from three independent experiments with at least three mice per time point. Friedman's test was used for statistical comparison. p.i., post-infection. ∗p < 0.05; ∗∗p < 0.01; ∗∗∗p < 0.001.

**Figure 2:**
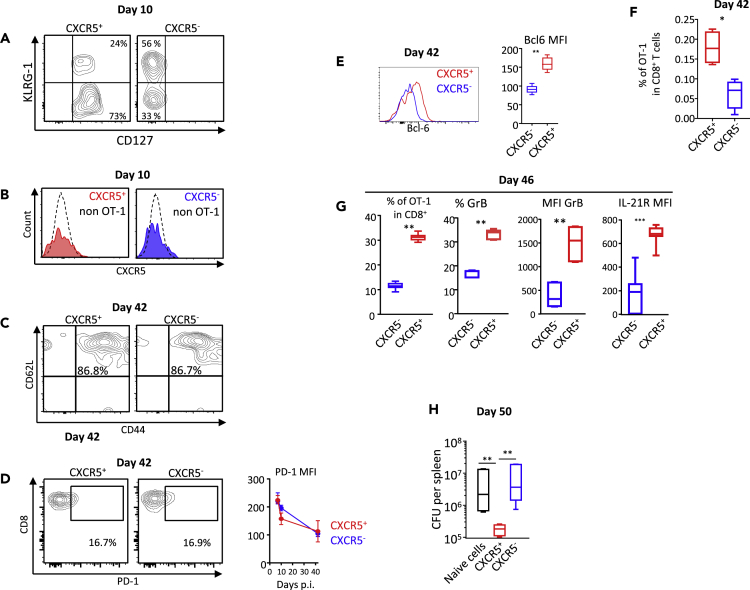
CXCR5^+^ Primary Effectors Differentiate into Highly Competent Memory Cells CXCR5^+^ or CXCR5^-^ OT-1 cells were sorted on day 3.5 after infection and transferred to recipient mice (see Figure S1 for the experimental design). The progeny of CXCR5^+^ or CXCR5^-^ OT-1 cells was examined at different time points after infection. (A) The expression of CD127 and KLRG-1 on day 10 post-infection by CXCR5^+^ and CXCR5^-^ OT-1. (B) The expression of CXCR5 on day 10 post-infection by CXCR5^+^ and CXCR5^-^ OT-1. (C) The expression of CD44 and CD62L by the progeny of CXCR5^+^ and CXCR5^-^ OT-1 cells, on day 42 post-infection, i.e., at the memory stage. (D) The expression of PD-1 by the progeny of CXCR5^+^ and CXCR5^-^ OT-1 cells, on day 42 post-infection, i.e., at the memory stage. PD-1 MFI are also shown from day 7 to day 42 post-infection. (E) The expression of Bcl-6 by the progeny of CXCR5^+^ and CXCR5^-^ OT-1 cells, on day 42 post-infection, i.e., at the memory stage. Bcl-6 MFI are also shown at day 42 post-infection. (F) The frequency in CD8^+^ T cells of OT-1 memory cells derived from the progeny of CXCR5+ or CXCR5- OT-1 cells. (G and H) Mice adoptively transferred with CXCR5^+^ or CXCR5^-^ OT-1 cells were challenged with rLm-OVA on day 42, i.e., at the memory stage. Owing to the possible presence of reacting endogenous memory CD8^+^ T cells, secondary OT-1 effectors were sorted 4 days after challenge (day 46) and injected into naive wild-type mice infected at the same time with 2 × 10^5^ colony-forming unit rLm-OVA. OT-1 cells and bacterial burden were analyzed 4 days after transfer (day 50, see Figures S1, 2G, and 2H). (G) Day 46, from left to right: the percentage in CD8^+^ T cells of OT-1 cells, the percentage of OT-1 cells expressing granzyme B, the corrected geometric MFI of granzyme B in OT-1 cells (expressed as [MFI^OT-1^ – MFI^non-OT-1^]), and the corrected geometric MFI of IL-21R in OT-1 cells (expressed as [MFI^OT-1^ – MFI^non-OT-1^]). (H) The bacterial burden measured in the spleen 4 days after infection (day 50). The data are from five independent experiments. Mann-Whitney test (E–G) and Kruskal-Wallis test (H) were used for statistical analysis. p.i., post-infection. ∗p < 0.05; ∗∗p < 0.01; ∗∗∗p < 0.001.

Recombinant *Listeria monocytogenes* (Lm)-OVA infection of naive mice led to strong expansion of OVA-specific CD8^+^ primary effectors, starting on day 3 and peaking on day 7, when they represented 42% of the total CD8^+^ T cells (Figure 1A). This CD8 expansion was associated with rapid control of bacterial multiplication in the spleen and liver, which became undetectable on day 7 after infection (Figure 1B). Expansion of OVA-specific CD8^+^ primary effectors was preceded by transient Tfh expansion (Figure 1C). Primary CD8^+^ effectors expressed CXCR5, the receptor for the chemokine CXCL13, as early as 2 days after priming (Figure 1D). CXCR5 expression within the pool of primary effectors was transient, peaking on day 3 and then rapidly declining to become barely detectable on day 6 (Figures 1D–1E). Based on CXCR5 expression, priming elicited two subsets of CD8^+^ effectors (Figure 1F). The CXCR5^+^ subset initially predominated within the pool of OVA-specific CD8^+^ effectors until day 4, before being overwhelmed by strong expansion of CXCR5^-^ cells and eventually becoming barely detectable (Figures 1D–1E and 1G). Phenotypic analysis showed that CXCR5^+^ and CXCR5^-^ effector CD8^+^ T cells expressed CD44 and similar levels of the effector marker KLRG-1, as well as PD-1 and the receptor of IL-21, with the exception of CD40, which was expressed at a higher level by CXCR5^+^ early CD8^+^ effectors (Figure 1H). Both subsets also down-regulated CD62L and CD127 (Figure 1H). We then examined the fate of CXCR5^+^ and CXCR5^-^ CD8^+^ early effectors and their ability to become memory cells, by means of adoptive transfer experiments on sorted cells (Figures S1 and 2). As shown in Figures 2A and 2B, at day 10 post-priming, most cells derived from CXCR5^+^ early effectors had lost CXCR5 and KLRG-1 expression and had become CD127^+^, whereas cells derived from CXCR5^-^ effectors were still CD127^-^, and half of them still expressed the effector marker KLRG-1 (Figure 2A). At day 42 post-priming, both cells derived from CXCR5^+^ and CXCR5^-^ early CD8 effectors had a central memory phenotype (CD44^+^CD62L^+^) and expressed similar low levels of PD-1 (Figures 2C and 2D); however, cells derived from CXCR5^+^ early CD8 effectors expressed higher levels of the memory pathway-associated transcription factor Bcl-6 (Figure 2E). Noteworthy, the frequency of the progeny of CXCR5^+^ early effectors in total CD8 T cells was higher than that of CXCR5^-^ early CD8 effectors, which may suggest better survival (Figure 2F). As shown in Figure 2G, following Lm-OVA re-infection, cells derived from CXCR5^+^ early CD8 effectors strongly expanded, expressed high levels of granzyme B and IL-21 receptor (Figure 2G), and were highly competent in controlling bacterial replication (Figure 2H), contrary to cells derived from CXCR5^-^ CD8^+^ early effectors (Figures 2G and 2H). Thus, a subset of CD8^+^ effectors expressing CXCR5 appears very early after antigen priming. This initially predominant subset rapidly becomes a minority subset among CD8^+^ primary effectors. Then those cells lose CXCR5 expression, exhibit phenotypic hallmarks of memory precursors cells (CD127^+^ KLRG-1^-^), and differentiate into highly functional memory cells. Altogether, this CXCR5^+^ early CD8 effector subset contains precursors of highly functional memory cells.

### CXCR5^+^ CD8^+^ Memory Precursors Interact with Tfh and B Cells at the T-B Cell Zone Junction

**Figure 3:**
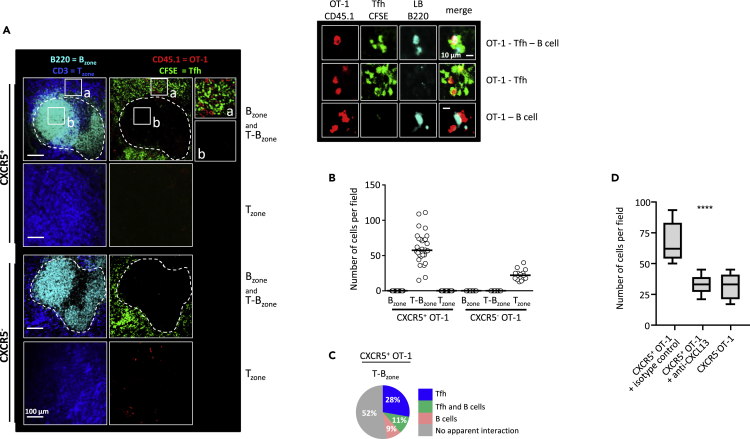
CXCR5^+^ Memory Precursors Interact with Tfh and B Cells at the T-B Cell Zone Junction Tfh CD4^+^ T cells, CXCR5^+^, and CXCR5^-^ OT-1 cells were sorted on day 3.5 post-priming and transferred (intravenously) to synchronously infected recipient mice. Sixteen hours later the mice were sacrificed, the spleens were removed, and cryosections were prepared for immunofluorescence staining and confocal imaging (see Figure S2 and Transparent Methods). (A and B) (A) Representative spleen sections after immunofluorescent staining. B cell zones are pseudo-colored in cyan, T cell zones in blue, Tfh CD4^+^ T cells in green, and transferred CXCR5^+^ and CXCR5^-^ OT-1 cells in red. The magnifications of areas inside the perifollicular zone (a) and B cell follicles (b) are shown. Higher-magnification views showing contacts between CXCR5^+^ OT-1 cells, Tfh, and B cells are also shown. Fields were randomly acquired for analysis (see Transparent Methods). B cell and T cell zones were identified by B220 and CD3 staining (see white dotted line). (B) Numbers of CXCR5^+^ and CXCR5^-^ OT-1 cells in B cell zones, perifollicular zones, and T cell zones. (C and D) (C) The percentage of interaction-free OT-1 cells and also the percentages of OT-1 cells in contact with Tfh and/or B cells, inside the T-B zone. The data are from three different experiments, each with at least 10 randomly imaged fields. (D) CXCR5^+^ and CXCR5^-^ OT-1 cells were sorted on day 3.5 post-priming and transferred intravenously to synchronously infected wild-type recipient mice. Some mice received an injection of a neutralizing antibody against CXCL13 or an isotype control (see Figure S2E). Mice were sacrificed 16 h later. Confocal microscopic analysis was performed on spleen cryosections. The numbers of CXCR5^+^ OT-1 cells and CXCR5^-^ OT-1 cells that reached the T-B junction zone are shown. Median and 10th and 90th percentiles of results obtained in 12 microscopic fields are shown. Statistical significance is indicated (****p < 0.0001, Kruskal-Wallis test).

Confocal microscopy (see Figure S2A and Transparent Methods) showed that, 4 days after priming, CXCR5^+^ OT-1 cells were exclusively located in the T-B cell zone junction (Figures 3A and 3B). By contrast, CXCR5^-^ OT-1 cells were exclusively located in T cell areas (Figure 3B), pointing to specific spatial distribution of CXCR5^+^ CD8^+^ OT-1 in the vicinity of B cell zones. These experiments were conducted after intravenous injection of sorted CXCR5^+^ and CXCR5^-^ OT-1 cells (see Transparent Methods and Figure S2A). Interestingly, CXCR5^+^ T cells migrated more efficiently to the spleen than did CXCR5^-^ T cells (Figure S2B). This may be related to the higher expression of CCR7 by CXCR5^+^ OT-1 cells (Figure S2C). Co-expression of CXCR5 and CCR7 may explain the localization of CXCR5^+^ OT-1 to the T-B border, as CCR7 expression can block T cell migration to the B cell zone (Haynes et al., 2007). Similar localization in T-B cell zone junctions has previously been reported for Tfh, which also express CXCR5 (Crotty, 2011, Vinuesa et al., 2016) (see Figure 3C and also Figures S2E and S2F that show localization at the T-B cell zone border of transferred and endogenous Tfh, respectively). Tfh and CXCR5^+^ OT-1 expressed similar levels of CXCR5 at the cell surface (Figure S2D). Neutralization of the ligand of CXCR5, the chemokine CXCL13, led to a sharp reduction in the number of CXCR5^+^ OT-1 cells in the T-B cell zone border (Figure 3D). This suggested that CXCR5 is required for the positioning of CD8 memory precursors at the junction between T and B cell zones. Moreover, 48% of CXCR5^+^ CD8^+^ MPEC colocalized with Tfh and/or B cells (28% and 9% with Tfh and B cells respectively; 11% with both Tfh and B cells; Figure 3C; see also Figure S2F that shows interactions of CXCR5^+^ OT-1 with endogenous Tfh). Together, these results suggest that, after priming, CXCR5^+^ OT-1 cells rapidly migrate from T cell areas to T-B cell zone junctions, where they may interact with Tfh and, to a lesser extent, with B cells.

### Tfh Are Required for Optimal Differentiation of CXCR5^+^ CD8^+^ Memory Precursors into Highly Functional Memory Cells

**Figure 4:**
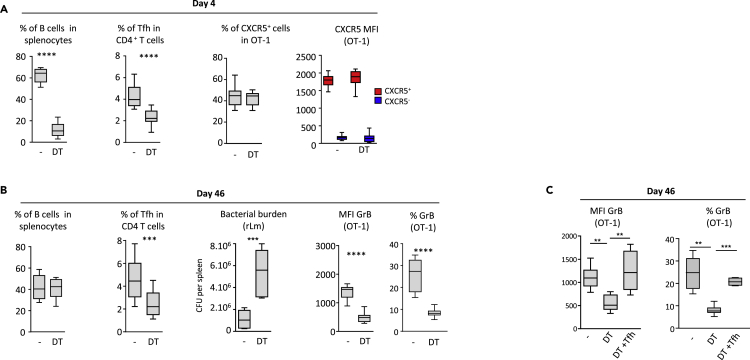
B Cells Help Generate Functional Memory CD8^+^ T Cells Indirectly, via Tfh CD19-diphtheria toxin receptor (DTR) mice received 10^4^ OT-1 cells and were then infected with 2 × 10^4^ colony-forming unit (CFU) of rLm-OVA. B cells were depleted with diphtheria toxin (DT) on days 1, 2, and 3 after infection. On day 42, mice were challenged with 2 × 10^5^ CFU of rLm-OVA, and secondary responses were evaluated 4 days later (see Figure S3 and Transparent Methods). (A) From left to right: B cell depletion, the percentage of Tfh among CD4^+^ T cells, the percentage of CXCR5-expressing OT-1 cells, and the geometric MFI of CXCR5 on OT-1 cells (analyzed as [MFI^OT-1^ – MFI^non-OT-1^]) in DT-treated and DT-untreated mice, on day 4 post-priming. (B and C) (B) From left to right, the percentage of B cells among splenocytes, the percentage of Tfh among CD4^+^ T cells, the splenic rLm-OVA burden, the corrected geometric MFI of granzyme B in OT-1 cells (expressed as [MFI^OT-1^ – MFI^non-OT−1^]), and the percentage of granzyme B-expressing OT-1 cells, 4 days after the challenge at the memory stage (day 46) among DT-treated and DT-untreated mice. (C) DT-treated CD19-DTR mice received adoptive transfer of sorted Tfh cells on day 4 post-priming (see Figure S3 and Transparent Methods). The mice were then challenged on day 42, and granzyme B expression by OT-1 cells was analyzed 4 days later (on day 46). The data shown in Figure 4 are the median and 10th and 90th percentiles of values from at least three to five independent experiments. Mann-Whitney (A and B) and Kruskal-Wallis (C) tests were used for statistical comparison. ∗∗p < 0.01; ∗∗∗p < 0.001; ∗∗∗∗p < 0.0001.

B cell depletion early after priming, starting 1 day after rLm-OVA infection (see Figures S3A and 4A), had no significant effect on the generation of CXCR5^+^ CD8^+^ OT-1 (Figure 4A). However, after rLm-OVA challenge, at the memory stage, when the B cell pool had been restored (Figure 4B), the memory CD8 response was impaired in terms of both *in vivo* bacterial control and granzyme B expression (Figure 4B). By contrast, the amplitude of secondary expansion was not affected (Figure S3B). Of interest, selective B cell depletion during priming affected both primary and secondary Tfh expansion (Figures 4A and 4B). This is consistent with previous studies showing the importance of B cells for generating and maintaining Tfh cells (Choi et al., 2011, Deenick et al., 2010). Reconstitution of the Tfh pool at the time of B cell depletion restored the cytotoxic functions of CD8^+^ memory cells (Figures S3A and 4C), pointing to an indirect effect of B cells on CD8 memory via their action on Tfh.

**Figure 5:**
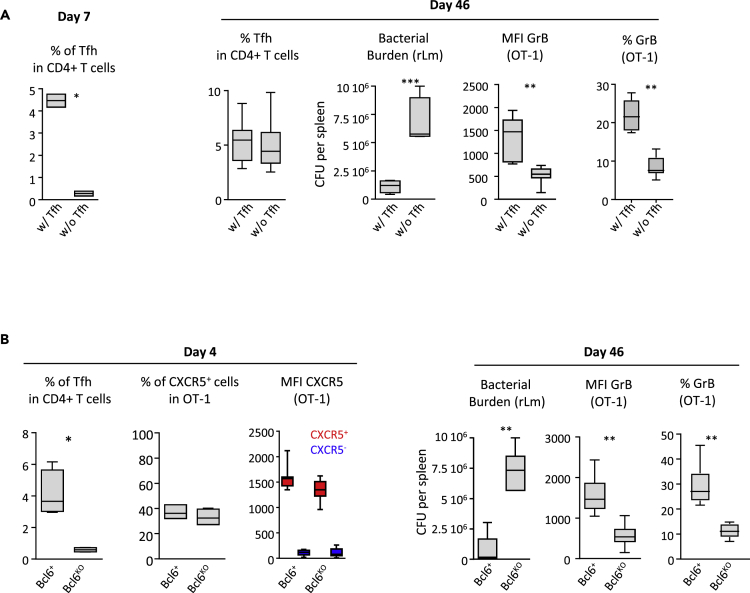
Tfh Are Required to Generate Functional CD8 Memory (A and B) (A) CD4-DTR mice received 10^4^ OT-1 cells and were then infected with 2 × 10^4^ colony-forming unit (CFU) of rLm-OVA. CD4^+^ cells were depleted with DT on days 1, 2, and 3 after infection. CD4-DTR mice were reconstituted on day 4 post-priming with total CD4^+^ T cells (w/Tfh) or with Tfh-depleted CD4^+^ T cells (w/o Tfh). On day 42, mice were challenged with 2 × 10^5^ CFU of rLm-OVA and secondary responses and bacterial burden were evaluated 4 days later (day 46) (see Figure S4A and Transparent Methods). (A) On the left, the frequency of Tfh among CD4^+^ T cells 3 days after transfer (day 7 post-infection) is shown. On the right, the percentage of Tfh cells among CD4^+^ T cells, the splenic rLm-OVA burden, the corrected geometric MFI of granzyme B in OT-1 cells (calculated as [MFI^OT-1^ – MFI^non-OT-1^]), and the percentage of granzyme B-positive OT-1 cells, 4 days after the challenge at the memory stage (day 46), are shown. (B) Similar experiments were conducted with tamoxifen-treated CD4-*bcl*6^floxed^ mice (Bcl-6^ko^ in the graph) or with tamoxifen-treated CD4-*bcl*6^+^ littermates (non-sensitive to tamoxifen, Bcl-6^+^ in the graph) (see Figure S4C and Transparent Methods). On the left, the frequency of Tfh in CD4^+^ T cells, the percentage of CXCR5^+^ cells among OT-1 cells, and the geometric corrected MFI of CXCR5 on OT-1 cells (as [MFI^OT-1^ – MFI^non-OT-1^]) were analyzed on day 4 post-infection. On the right, the splenic rLm-OVA burden after challenge, the corrected geometric MFI of granzyme B in OT-1 cells (calculated as [MFI^OT-1^ – MFI^non-OT-1^]), and the percentage of granzyme B-positive OT-1 cells after challenge (day 46) are shown. Graphs display median and 10th and 90th percentiles. Mann-Whitney test was used for statistical comparison. ∗p < 0.05; ∗∗p < 0.01; ∗∗∗p < 0.001.

**Figure 6:**
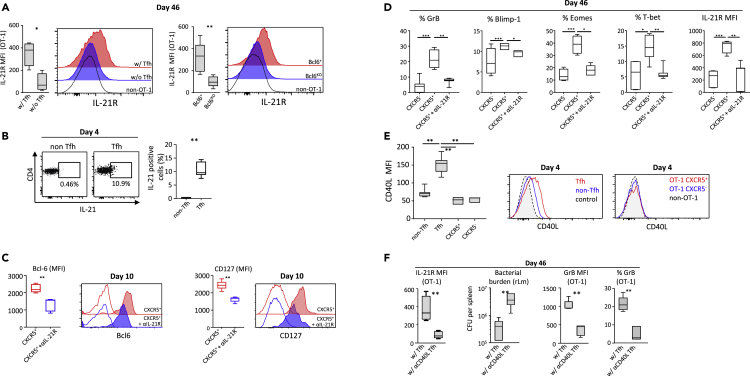
Tfh Provide Help through IL-21 and CD40 Ligation (A) IL-21R expression by the secondary effectors (4 days after challenge, at day 46) generated in the two experimental models of Tfh depletion shown in Figure 5 (see also Figures S4A and S4C). The results are expressed as corrected geometric MFI, i.e., [MFI^OT-1^ – MFI^non-OT-1^]. Representative plot profiles are also shown. (B and C) (B) IL-21 expression by Tfh and non-Tfh CD4^+^ T cells, at day 4 post-infection (p.i.). (C) CXCR5^+^ OT-1 cells sorted at day 3.5 p.i. were treated with anti-IL-21R (+αIL-21R, in blue) or with anti-IL-21R isotype control (in red) and were adoptively transferred into recipient mice (see Figure S5 and Transparent Methods). Bcl6 and CD127 expression, at day 10 p.i., are shown in both conditions. Isotype control staining (open histograms) is also shown. (D) OT-1 memory response was analyzed 4 days after the challenge at the memory stage (day 46). Percentages of granzyme B, Blimp-1, eomesodermin- and T-bet-positive cells, and corrected geometric MFI of IL-21R (i.e., [MFI^OT-1^ – MFI^non-OT-1^]) in secondary effectors derived from CXCR5^-^ OT-1 cells, anti-IL21R-treated CXCR5^+^ OT-1 cells, or isotype control-treated CXCR5^+^ OT-1 cells are shown. (E and F) (E) At day 4 p.i., geometric MFI of CD40 ligand at the cell surface of Tfh and CXCR5^+^ or CXCR5^-^ OT-1 primary effectors. Representative plot profiles are also shown. (F) CD4-DTR mice received 10^4^ OT-1 cells and were then infected with 2 × 10^4^ colony-forming unit (CFU) of rLm-OVA. CD4^+^ T cells were depleted with DT on days 1, 2, and 3 after infection. CD4-DTR mice were reconstituted on day 4 post-priming with Tfh-depleted CD4^+^ T cells and either Tfh treated with neutralizing anti-CD40L (w/αCD40L Tfh) or Tfh treated with the corresponding isotype control (w/Tfh). On day 42, mice were challenged with 2 × 10^5^ CFU of rLm-OVA, and secondary responses and bacterial burden were evaluated 4 days later (day 46) (see Figure S6 and Transparent Methods). Data shown are, from left to right, corrected geometric MFI of IL-21R at the cell surface of OT-1 cells (calculated as [MFI^OT-1^ – MFI^non-OT-1^]), splenic rLm-OVA burden, granzyme B-corrected geometric MFI in OT-1 cells (calculated as [MFI^OT-1^ – MFI^non-OT-1^]), and the percentage of granzyme B-positive OT-1 cells. The data shown in Figure 6 are the median and 10^th^ and 90^th^ percentiles of values obtained in three independent experiments with at least three mice per condition. Data from (A, C, and F) were analyzed with the Mann-Whitney test, data from (B) with the Wilcoxon test, data from (D) with the Kruskal-Wallis test, and data from (E) with the Friedman test. *p < 0.05; **p < 0.01; ***p < 0.001.

Next, we examined the influence of Tfh depletion on CXCR5^+^ CD8^+^ OT-1 in two experimental models of selective Tfh depletion early after priming (see Figures S4, 5A, and 5B). Like B cell depletion, Tfh depletion had no significant effect on the generation of CXCR5^+^ CD8^+^ OT-1 (Figure 5B and data not shown). However, at the memory stage, whereas CD8 secondary expansion was not affected after bacterial challenge (Figure S4B), *in vivo* bacterial control and granzyme B expression were both severely impaired (Figures 5A and 5B). Strikingly, in the absence of Tfh during priming, CD8^+^ memory T cells lacked the ability to up-regulate IL-21R after challenge with rLm-OVA (Figure 6A), whereas IL-21 was critically involved in effector functions of memory CD8^+^ T cells (Elsaesser et al., 2009, Fröhlich et al., 2009, Tian and Zajac, 2016, Yi et al., 2009).

IL-21 may also help for the differentiation of CD8 memory precursors as previously reported (Cui et al., 2011). IL-21 is produced by Tfh (Crotty, 2011, Vinuesa et al., 2016, and Figure 6B). In CXCR5^+^ CD8 memory precursors, IL-21, *in vivo*, up-regulated expression of both the transcriptional factor Bcl6 and the alpha subunit of IL-7 receptor (Figures S5 and 6C). Bcl-6 and IL-7Rα are involved in CD8 memory precursors differentiation (Crotty et al., 2010, Huster et al., 2004, Kaech et al., 2003). Neutralization of IL-21R on CXCR5^+^ OT-1 memory precursors altered the ability of the derived memory cells to up-regulate, following antigen activation, eomesodermin, T-bet, and Blimp-1, three critical transcription factors associated with effector differentiation (Kaech and Cui, 2012), as well as the expression of granzyme B and IL-21R (Figure 6D). This suggested a helping role of IL-21 in the differentiation of CXCR5^+^ CD8 memory precursors and confirmed a differential effect of this cytokine on CD8 T cells according to their state of differentiation.

CD40 signaling provided by Tfh is critical for B cell memory differentiation (Vinuesa et al., 2016, Crotty, 2011). We found that CXCR5^+^ CD8 memory precursors expressed CD40 (Figure 1H), but not CD40 ligand (Figure 6E), at the time of their encounter with Tfh. CXCR5^-^ early CD8^+^ effectors also expressed CD40, but at a lower level than CXCR5^+^CD8 memory precursors (Figure 1H), whereas Tfh expressed CD40 ligand (Figure 6E). Blockade of CD40 ligand at the surface of Tfh at the time of CXCR5^+^CD8 memory precursors and Tfh interaction abrogated the positive effects of Tfh on the generation of a functional CD8 memory in terms of IL-21R up-regulation, granzyme B expression, and *in vivo* bacterial control following bacterial challenge (Figures S6 and 6F). Together, the results show that Tfh critically help CXCR5^+^ CD8 memory precursors to become highly functional memory cells through a set of signals that include CD40 ligation and IL-21.

## Discussion

Here, we identified CXCR5 as a very early marker of the CD8 memory differentiation pathway. Early CXCR5^+^ effectors with memory potential have an effector phenotype similar to that of CXCR5^-^ future short-lived primary effectors. One possible interpretation is that, starting from a common initial effector status that immediately follows antigen activation of the naive cell, the pathways that generate memory cells versus short-lived primary effectors bifurcate very early (Figure S7). Memory cells therefore appear to derive from a subset of very early effectors. This is in line with recent observations that CD8^+^ effectors may dedifferentiate into long-lived memory cells (Youngblood et al., 2017). CXCR5 may be valuable as a very early marker of the CD8 memory pathway in future studies of molecular events involved in memory differentiation. The CXCR5-CXCL13 signaling axis appears required for the transient positioning of CD8 memory precursors at the T-B cell zone junction. This is in contrast with a previous report suggesting that CXCR5 may not be essential for antigen-engaged CD4 T cells to localize to the T-B boundary (Haynes et al., 2007). CD8^+^ memory cells (and subsequently secondary effectors) derived from CXCR5^+^ memory precursors do not express CXCR5 (Figure S8A), although we cannot rule out the possibility that further differentiation leads some memory or secondary effector subsets to re-express CXCR5 in particular situations, as recently described in a context of chronic viral infections (He et al., 2016, Im et al., 2016). CXCR5^-^ and CXCR5^+^ early CD8^+^ effectors expand successively, with a temporal shift and different amplitudes. A first wave of expansion involves CXCR5^+^ effectors with memory potential, which transiently become predominant. Massive proliferation of CXCR5^-^ short-lived primary effectors occurs a few days later, overwhelming memory precursors (Figure S7). This time shift may reflect the need for a period of relative immunological calm that could favor early CXCR5^+^ memory precursor maturation events, including migration toward B-T cell zone junctions and cell-cell interactions. This is in line with the observation that excessive exposure to IL-2 and pro-inflammatory cytokines can impair CD8 memory development (Kalia et al., 2010, Pipkin et al., 2010). Also, the proliferation of CXCR5^+^ MPEC might be gradually hindered as the cells progress along a memory differentiation pathway, leading to further gradual dilution of these cells within the growing mass of short-lived primary effectors.

Another major finding is that help from Tfh, a specialized CD4^+^ subset until now considered as exclusive B cell helpers, is required for optimal development of CXCR5^+^ early memory precursors into highly functional memory cells. B cells also influence memory CD8 differentiation through their effects on Tfh. Without help signals from Tfh, memory CD8^+^ T cells appear unable to mount efficient secondary cytotoxic responses. This is not related to higher expression of exhaustion-associated inhibitory receptors such as PD-1 (Figures 2D and S8B). Instead, in the absence of help signals from Tfh, generated memory CD8^+^ T cells lack the ability to up-regulate IL-21R following antigen reactivation. IL-21 has been shown to enhance and sustain effector functions of memory cells in various models of chronic viral infections (Elsaesser et al., 2009, Fröhlich et al., 2009, Tian and Zajac, 2016, Yi et al., 2009). Our results suggest that the positive effect of Tfh on the generation of CD8 memory may involve IL-21. Indeed, blockade of this cytokine receptor on CXCR5+ CD8 memory precursors, at the time of their interaction with Tfh, impairs induction of Bcl6 and the alpha subunit of the IL-7 receptor, as well as the ability of the derived memory cells to up-regulate, following challenge, IL-21 receptor and key transcription factors involved in effector differentiation, including Blimp-1, T-bet, and eomesodermin (Figure 6D). These results suggest that IL-21 may favor differentiation of CXCR5^+^ memory precursors and also point out a differential effect of IL-21 according the stage of differentiation of CD8 T cells. This is in line with a previous report showing a positive effect of IL-21 on the generation of CD8 memory (Cui et al., 2011).

Another key point is that CXCR5^+^ memory precursors express CD40 and that blockade of CD40-CD40 ligand interaction abrogated Tfh-mediated help. CD40 expression has been already reported in activated CD8 T cells (Bourgeois et al., 2002). Help provided by Tfh to CXCR5^+^ memory precursors may therefore include CD40 ligation, possibly through direct interaction, similarly to B cells, although we cannot rule out the involvement of additional cell players such as dendritic cells in the process of Tfh-mediated help. CXCR5^+^ memory precursors therefore encounter Tfh at T-B cell boundaries and imprint them, through a panel of signals that may include CD40 ligation and IL-21, with an instructional program that enables memory cells to up-regulate IL-21 receptor following antigen reactivation and to become efficient killer cells.

We found that (1) CD8^+^ early memory precursors move at the T-B cell zone junction where B cells that have bound antigen first engage in cognate interactions with T cells to receive help, (2) the same CD4^+^ T cell subset (Tfh) controls the development of both CD8 and B cell memory pathways, and (3) B cells also influence the generation of CD8 memory, indirectly, through their effects on the generation and maintenance of Tfh. This suggests early coordination between the pathways of CD8 and B cell memory generation, through Tfh (See Figure S9). These results may have implications for vaccine and immunotherapy design.

### Limitations of the Study

We used cell sorting and adoptive transfer procedures that may have an impact on cells.

The subset(s) of Tfh that provide(s) help to CXCR5^+^ CD8 memory precursors (MPEC), and their fate following interaction with MPEC, remain to be clarified.

## Methods

All methods can be found in the accompanying Transparent Methods supplemental file.

## Supplemental Information

Document S1. Transparent Methods and Figures S1–S9
