# Supplementary material for: Follicular CD4 T Cells Tutor CD8 Early Memory Precursors: An Initiatory Journey to the Frontier of B Cell Territory
Source: iScience. 2019 Sep 14;20:100–9. doi: 10.1016/j.isci.2019.09.012 (PMC6817688; doi:10.1016/j.isci.2019.09.012)
Supplement: Document S1. Transparent Methods and Figures S1–S9 [file mmc1.pdf]

ISCI, Volume *20*

Supplemental Information

Follicular CD4 T Cells Tutor CD8 Early Memory Precursors: An Initiatory Journey to the Frontier of B Cell Territory

Marie-Ghislaine de Goër de Herve, Maïsa Abdoh, Salma Jaafoura, Deniz Durali, and Yassine Taoufik

SUPPLEMENTAL INFORMATION

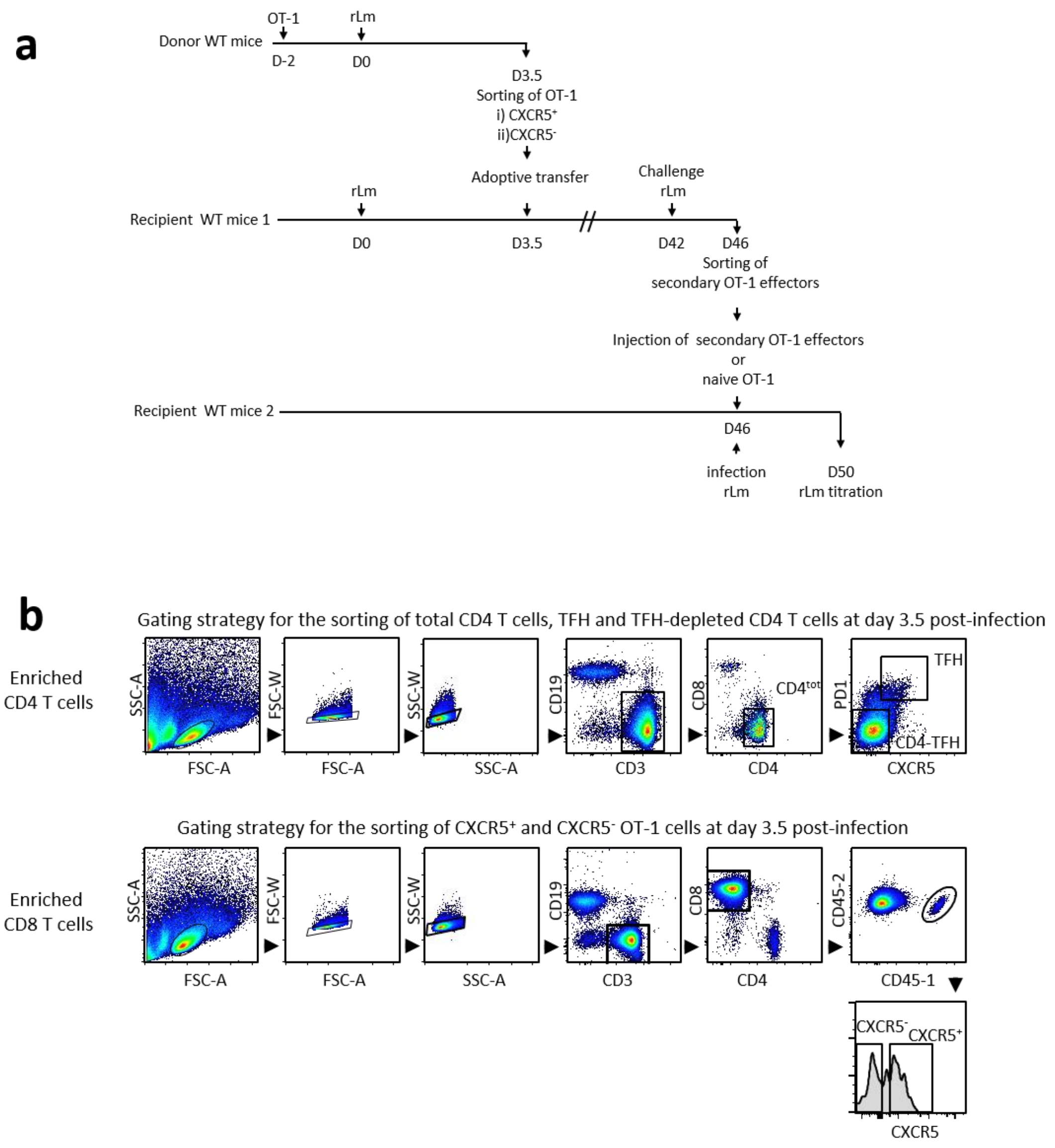

Figure S1. **CXCR5^{+} and CXCR5^{-} OT-1 sorting and adoptive transfer experiments (Related to Figure 2)**

Fig. S1a: WT C57BL6/J mice received 10^{4} OT-1 CD45.1^{+} cells before infection with 2.10^{4} CFU of rLm-OVA. Primary effector OT-1 cells (CXCR5^{+} or CXCR5^{-}) were sorted at the time of the peak of CXCR5 expression, i.e. 3.5 days post-priming, and were then injected i.v. into synchronously infected WT recipient mice. Recipient mice were then challenged on day 42 with 2.10^{5} CFU of rLm-OVA. Owing to the possible presence of reacting endogenous memory CD8^{+} T cells, secondary OT-1 effectors were sorted 4 days after challenge (at day 46) and injected into a second set of naive WT mice infected at the same time with 2.10^{5} CFU rLm-OVA. Control mice received the same number of naive OT-1 CD8^{+} T cells.

Fig. S1b shows the gating strategy for cell sorting of purified CXCR5^{+} OT-1, CXCR5^{-} OT-1, CD4^{+} T cells or Tfh cells. Enriched CD8^{+} T cells or enriched CD4^{+} T cells were obtained as described in the supplementary methods. Doublets were excluded on the basis of both forward scatter (FSC) and side scatter (SSC). CXCR5^{+} and CXCR5^{-} OT-1 cells were sorted in the CD19^{-} CD3^{+} CD4^{-} CD8^{+} CD45.2^{+} CD45.1^{+} cell gate. Highly purified total CD4^{+} T cells were sorted from the CD19^{-} CD3^{+} CD8^{-} CD4^{+} cell gate. Tfh-depleted CD4^{+} T cells were defined as CD19^{-} CD3^{+} CD8^{-} CD4^{+} PD-1^{-} CXCR5^{-} cells and Tfh as CD19^{-} CD3^{+} CD8^{-} CD4^{+} CXCR5^{+} PD-1^{hi}.

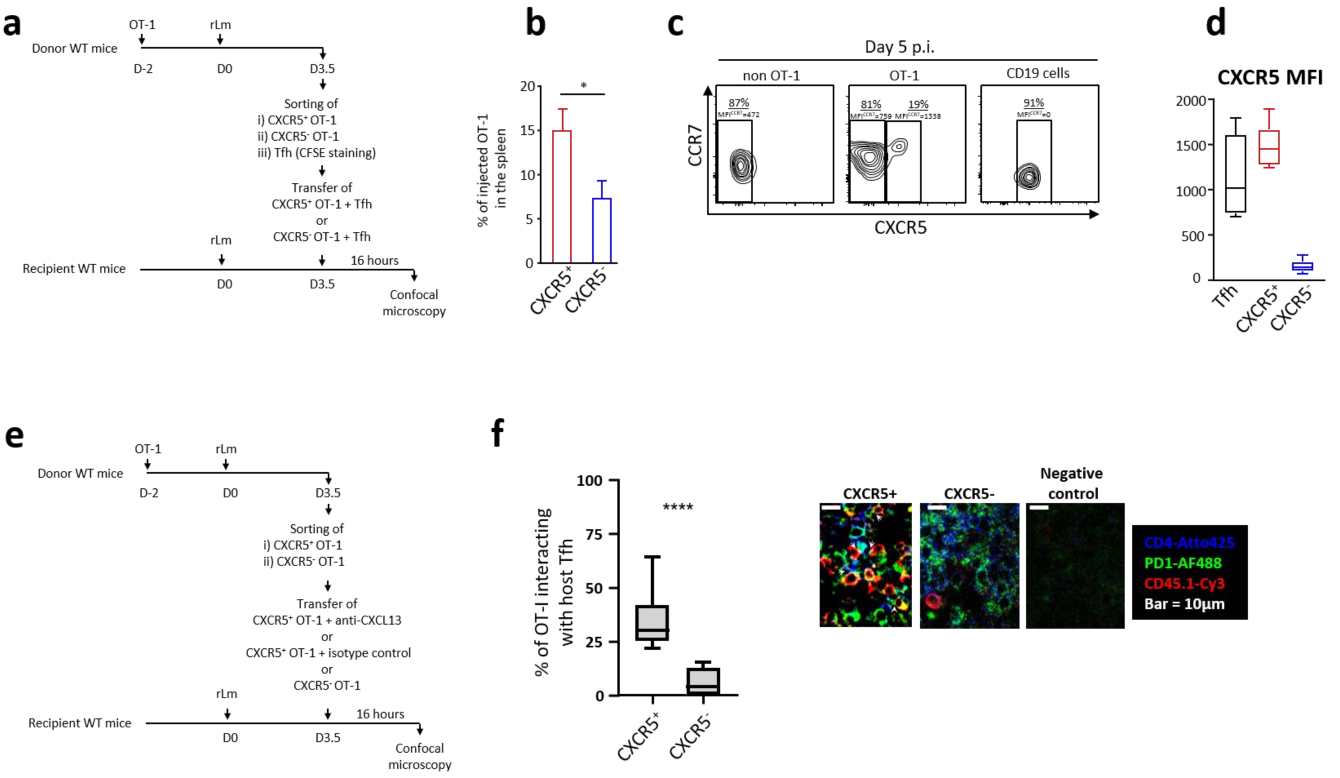

Figure S2. Confocal microscopy experiments (Related to Figure 3)

Fig. S2a. WT C57BL6/J mice received 10^6 OT-1 CD45.1$^+$ cells and were then infected with 2.10^4 CFU of rLm-OVA. Tfh, CXCR5$^+$ and CXCR5$^-$ OT-1 cells were sorted on day 3.5 post-priming. Cells were injected i.v. into synchronously infected recipient mice. Sixteen hours later the mice were sacrificed, the spleens were removed, and cryosections were prepared for immunofluorescence staining and confocal imaging (see Methods).

Fig. S2b shows the percentage of injected CXCR5$^+$ or CXCR5$^-$ OT-1 cells that reached the spleen 16 hours after injection (see methods). The Mann-Whitney test was used for statistical comparison.

Fig. S2c shows CXCR5 and CCR7 expression by non OT-1 CD8$^+$ T cells, OT-1 CD8$^+$ T cells, and CD19 cells as a control, on day 5 post-infection. Geometric MFI of CCR7 are indicated.

Fig. S2d shows the geometric MFI of CXCR5 at the cell surface of Tfh and CXCR5$^+$ and CXCR5$^-$ OT-1 cells.

Fig. S2e: WT C57BL6/J mice received 10^6 OT-1 CD45.1$^+$ cells and were then infected with 2.10^4 CFU of rLm-OVA. CXCR5$^+$ and CXCR5$^-$ OT-1 cells were sorted on day 3.5 post-priming and transferred i.v. to synchronously infected wild-type recipient mice. Mice were sacrificed 16 hours later. Confocal microscopy analysis was performed on spleen cryosections.

Fig. S2f: The percentage of adoptively transferred CXCR5$^+$ or CXCR5$^-$ OT-1 cells, that interact with endogenous Tfh from the recipient mice are shown. Median, 10th and 90th percentiles of results obtained in 12 microscopic fields are shown. Statistical significance is indicated (****, $p<0.0001$, Mann-Whitney test). Representative confocal microscopy images are also shown. Endogenous Tfh are detected via CD4 (blue) and PD-1 (green) staining. OT-1 are detected by CD45.1 staining (red). Interactions between OT-1 and endogenous TFH are indicated by arrowheads. Control with staining with only secondary antibodies is also shown. White bar represents 10 µm scale bar.

a

b

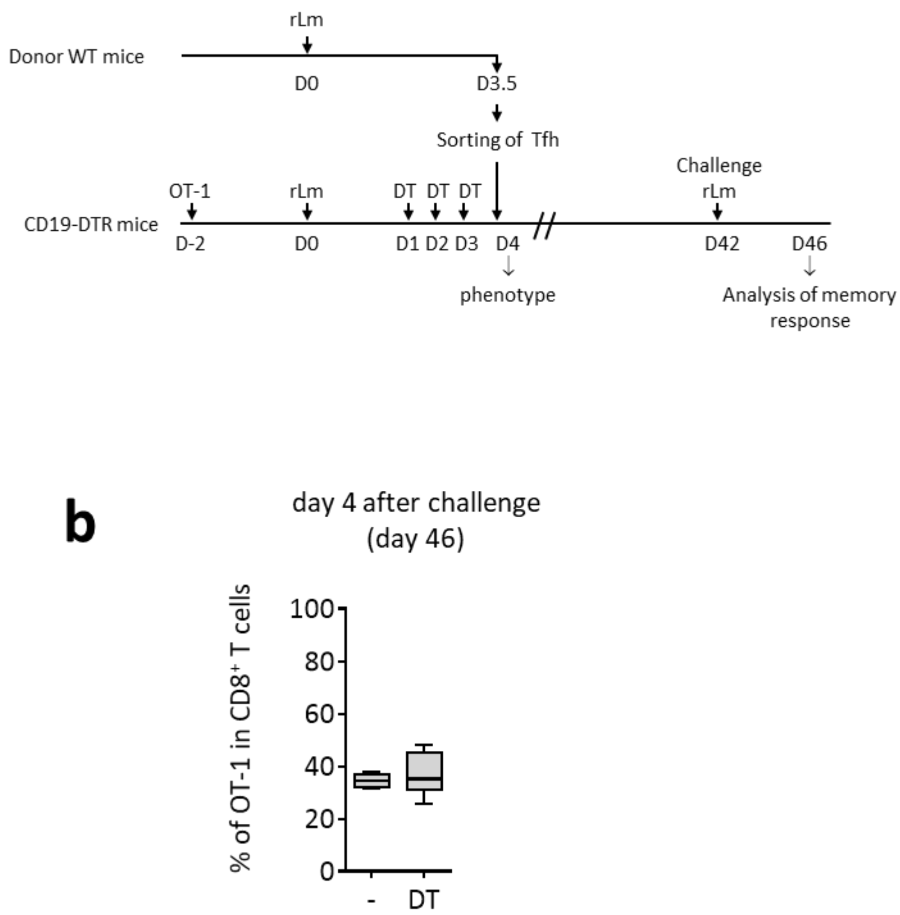

Figure S3. B cell depletion experiments (Related to Figure 4)

Fig. S3a. CD19-DTR mice received 10^4 OT-1 cells before infection with 2.10^4 CFU of rLm-OVA. The mice then received 3 injections of DT, on days 1, 2 and 3 post-priming to deplete B cells. In some experiments, mice received on day 3.5 sorted Tfh $CD4^+$ T cells obtained from a synchronously infected WT donor mouse. On day 42, the mice were challenged with 2.10^5 CFU of rLm-OVA and secondary responses and bacterial burden were examined 4 days later.

Fig. S3b shows secondary expansion of memory OT-1 cells after challenge (day 46) in mice depleted of B cells at the time of priming.

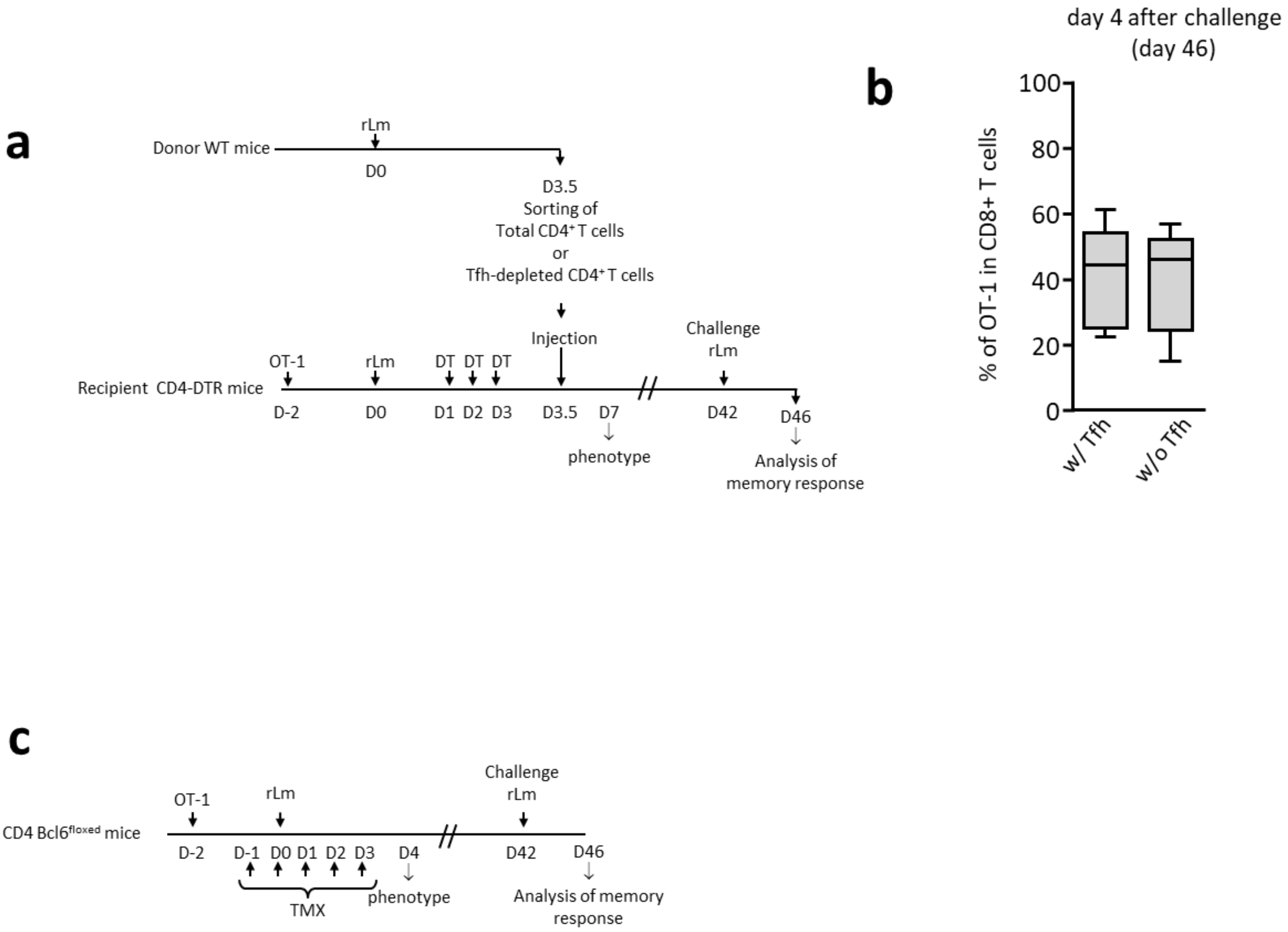

Figure S4. Tfh depletion experiments (Related to Figure 5 and Figure 6)

Fig. S4a. CD4-DTR mice received 10^4 OT-1 CD45.1$^+$ cells before infection with 2.10^4 CFU of rLm-OVA. CD4$^+$ T cells were depleted by DT injection on days 1, 2 and 3. On day 3.5 post-priming, mice received adoptive transfer of total CD4$^+$ T cells or Tfh-depleted CD4$^+$ T cells obtained from a synchronously infected WT donor mouse. On day 42, mice were challenged with 2.10^5 CFU of rLm-OVA. Secondary responses and bacterial burden were examined 4 days later.

Fig. S4b shows secondary expansion of OT-1 memory cells after challenge (day 46).

Fig. S4c: CD4-Bcl6^{floxed} or CD4-Bcl6$^+$ mice (see methods) received 10^4 OT-1 CD8$^+$ T cells before infection with 2.10^4 CFU of rLm-OVA. To deplete CD4$^+$ T cells expressing Bcl-6, mice received a daily tamoxifen injection for 5 days (day -1 to day 3). On day 42, the mice were challenged with 2.10^5 CFU of rLm-OVA. Secondary responses and bacterial burden were examined 4 days later (day 46).

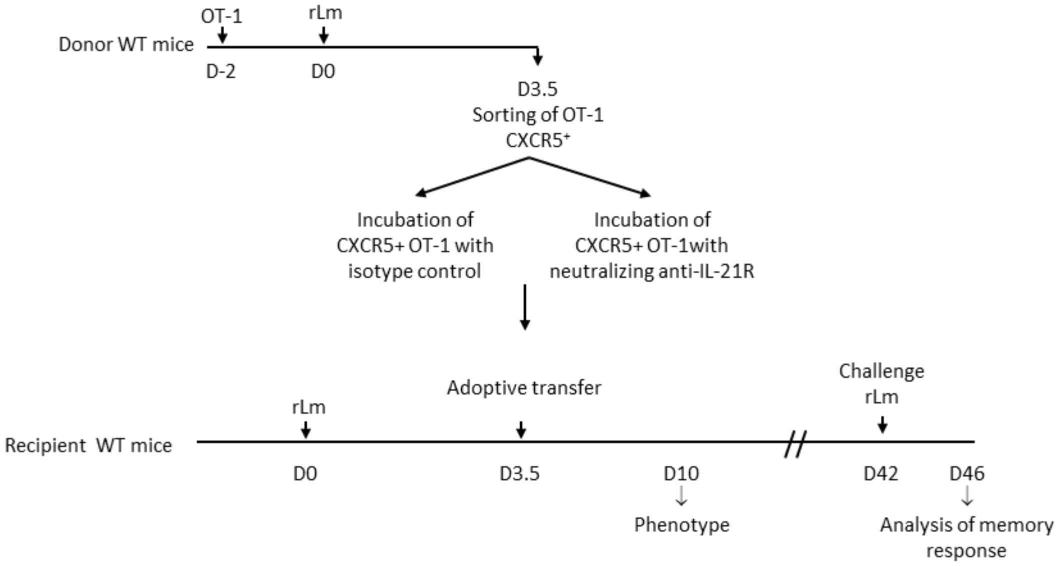

Figure S5. Blockade of IL-21 signaling during CD8 memory differentiation (Related to Figure 6)

WT C57BL6/J mice received 10^4 OT-1 CD45.1$^+$ cells before infection with 2.10^4 CFU of rLm-OVA. CXCR5$^+$ OT-1 cells were sorted at the time of the peak of CXCR5 expression, i.e. 3.5 days post-priming and incubated with neutralizing anti-IL-21R (αIL-21R) or the corresponding isotype control. Cells were then injected i.v. into synchronously infected WT recipient mice. OT-1 cells were analyzed at day 10 post-infection. In some experiments, mice were challenged on day 42 with 2.10^5 CFU of rLm-OVA. Secondary responses and bacterial burden were examined 4 days later (day 46).

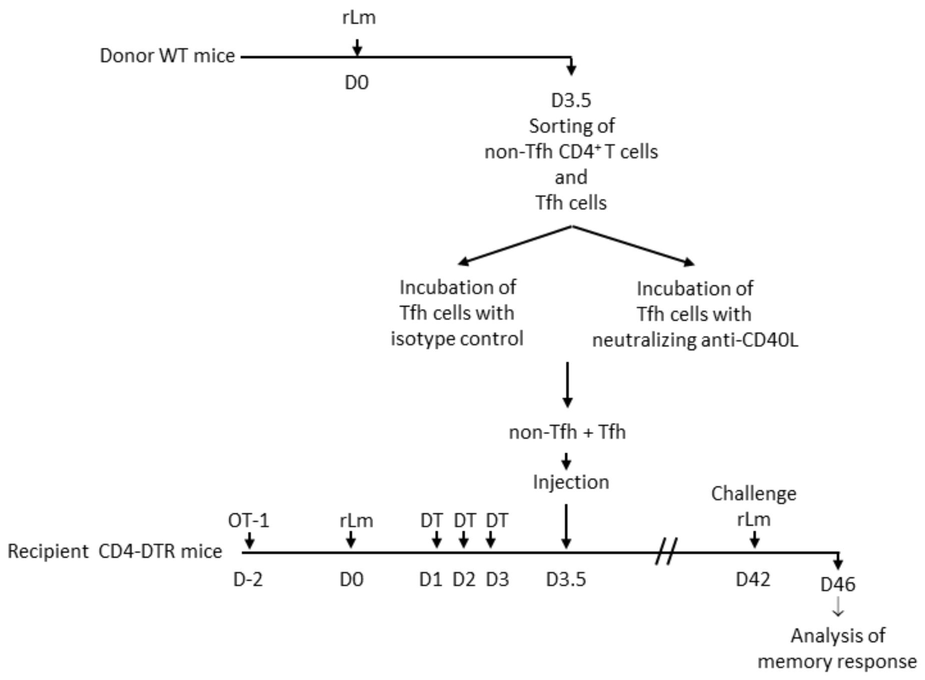

Figure S6. CD40L neutralization on Tfh (Related to Figure 6)

CD4-DTR mice received 10^4 OT-1 CD45.1$^+$ cells before infection with 2.10^4 CFU of rLm-OVA. CD4$^+$ T cells were depleted by DT injection on days 1, 2 and 3. On day 3.5 post-priming, mice were adoptively transferred with 1) non-Tfh CD4$^+$ T cells and neutralizing anti-CD40L-treated Tfh cells; or 2) non-Tfh CD4$^+$ T cells and isotype control-treated Tfh cells, obtained from synchronously infected WT donor mice. On day 42, mice were challenged with 2.10^5 CFU of rLm-OVA. Secondary responses and bacterial burden were examined 4 days later (day 46).

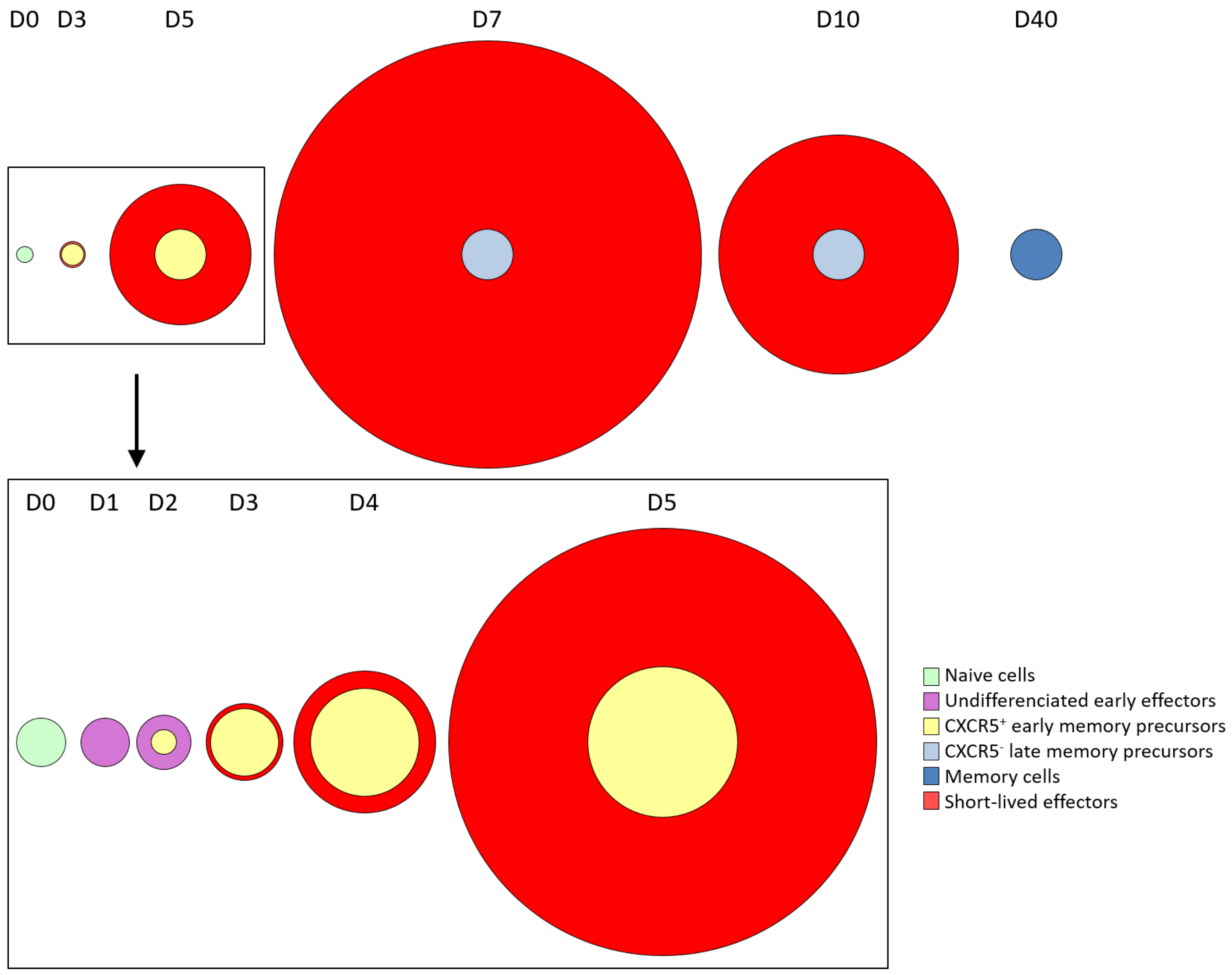

Figure S7. Dynamics of memory precursors and short lived effectors following antigen priming (Related to Discussion)

$CXCR5^+$ memory precursors and $CXCR5^-$ short lived effectors appear from a common initial effector, and expand successively with a temporal shift and different amplitudes. A first wave of expansion involves $CXCR5^+$ memory precursors which transiently become predominant. A few days later, memory precursors downregulate CXCR5, while strong proliferation of $CXCR5^-$ short lived effectors occurs, overwhelming memory precursors, before to undergo massive apoptosis. The pool of memory precursors remains stable and pursues maturation to become highly functional memory cells. The surface areas are proportional to the numbers of cells in 10^6 $CD8^+$ T cells.

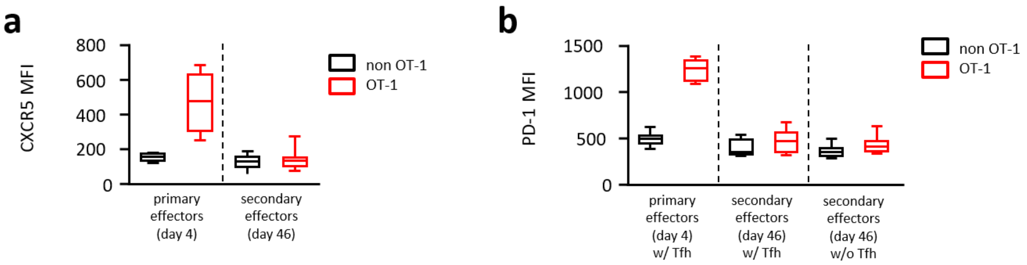

Figure S8. Secondary CD8^{+} effectors generated in the presence or absence of Tfh do not express CXCR5 nor PD-1 (Related to Discussion)

Fig. S8a: CXCR5 expression was analyzed at the cell surface of primary (at day 4) or secondary (at day 46) CD8 effectors**.**

Fig. S8b: PD-1 expression was analyzed at the cell surface of primary (at day 4) or secondary (at day 46) CD8 effectors, generated in CD4-DTR mice adoptively transferred with total CD4+ T cells (w/ Tfh) or with Tfh-depleted CD4^{+} T cells (w/o Tfh) (See **Fig. S4a**).

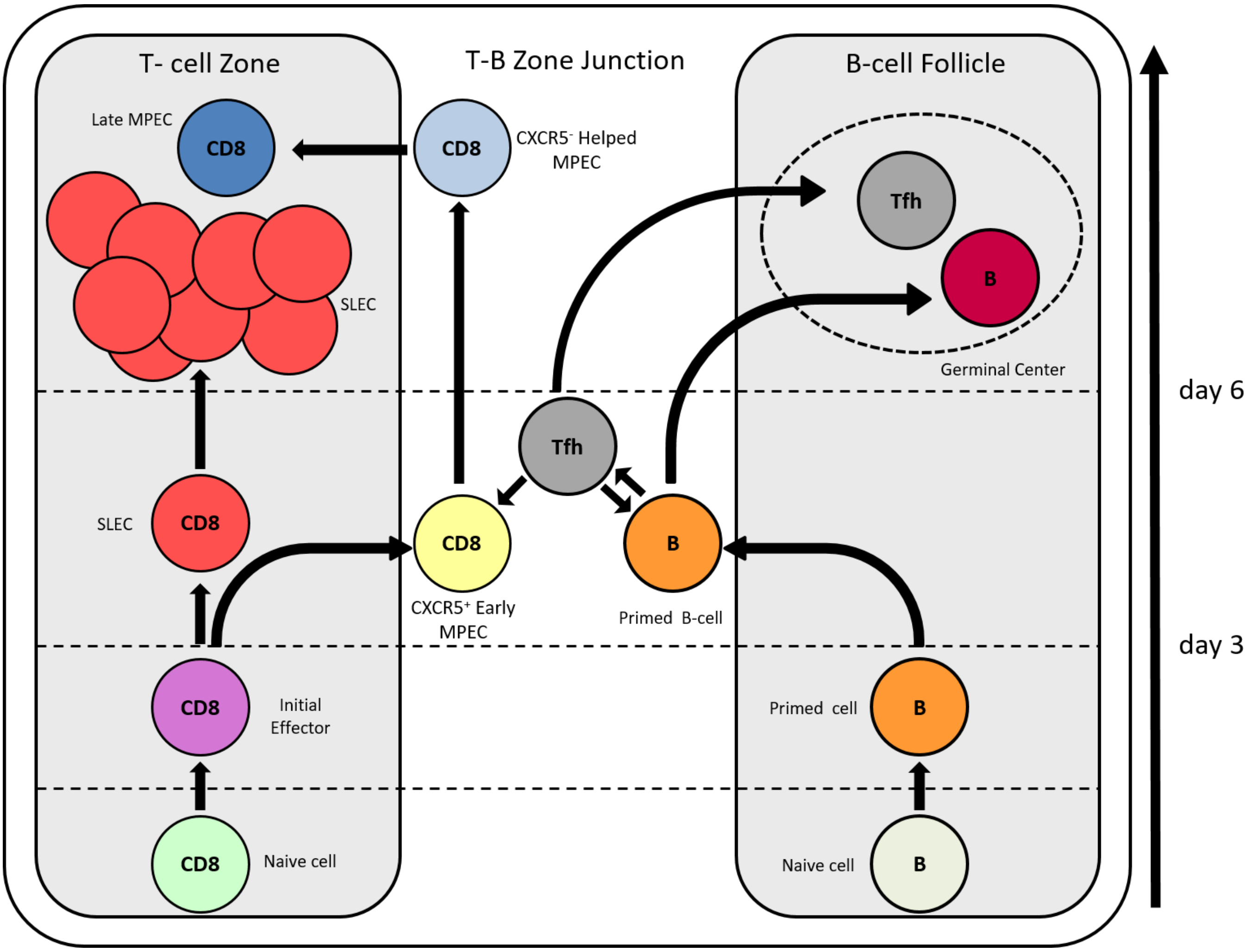

Figure S9. Possible Coordination of CD8$^+$ T cell and B cell Memory Differentiation Pathways through Tfh (Related to Discussion)

In the T-cell zone, starting from a common initial effector status that follows antigen activation of the CD8$^+$ naive cell, the pathways that generate memory cells versus short-lived primary effectors bifurcate very early. Early memory precursors express CXCR5, expand then migrate to the T-B cell junction zone where they receive helping signals from Tfh. Antigen-primed B cells also receive help from Tfh at the T-B cell junction, through cognate interactions. Those cell interactions with Tfh could be exclusive (B cell and CD8$^+$ memory precursors interact with distinct cells or subsets of Tfh), sequential (B cell and CD8$^+$ memory precursors interact successively with the same Tfh) or simultaneous (triple cell interactions). Indeed, confocal microscopy experiments showed triple colocalization, raising the possibility of triple cell interactions (See **Fig. 3**). CD8$^+$ memory precursors then downregulate CXCR5 and return to T-cell areas, where they pursue memory differentiation, while Tfh and B-cell relocate in the germinal center. In T cell areas, CXCR5$^-$ CD8$^+$ short lived-effectors strongly expand, with a temporal shift as compared to CXCR5$^+$ CD8$^+$ memory precursors, before to undergo massive apoptosis.

TRANSPARENT METHODS

Mice and infection

All mouse experiments were approved by local ethics committees (CEEA n°26 for projects 2012-086 and 7571; and CEEA n°5 for project 9143). All mice had the C57BL/6 background. Naive mice were housed in a specific-pathogen-free animal care facility (Faculty of Medicine Paris-Sud, Le Kremlin-Bicêtre). WT C57BL/6 mice were purchased from Janvier (Le Genest-Saint-Isle, France). OT-1 and CD45-1 mice were purchased from Charles River Laboratories (L'Arbresles, France). CD45-1-expressing OT-1 cells were generated by mating OT-1 mice with CD45-1 mice.
CD19-DTR mice were generated by mating CD19-cre mice (Cd19^{tm1}(cre) Cgn/J, SN6785) (Rickert et al., 1997) and iDTR mice (C57BL/6-Gt(ROSA)26Sortm1(HBEGF)Awai/J, SN7900). (Buch et al., 2005).
CD4-DTR mice were generated by mating CD4-cre mice (Lee et al., 2001) (B6.Cg-Tg(Cd4-cre)1Cwi/BfluJ, SN22071) with iDTR mice.
CD4-bcl6^{floxed} mice were generated by mating CD4-cre^{ERT2} mice (Aghajani et al., 2012) (B6(129X1)-Tg (Cd4-cre/ERT2)11Gnri/J, SN22356), and *Bcl6$^{fl/fl}$* mice (Hollister et al., 2013) (B6.129S(FVB)-Bcl6tm1.1Dent/J, SN23727). CD4-bcl6^{+} littermates were used as controls.
All these mice were purchased from Jackson Laboratories. Mice were genotyped with Jackson protocols.
CD19-DTR mice and CD4-DTR mice received diphtheria toxin (DT) for 3 days by intraperitoneal injection (1 µg per day; Sigma Aldrich). CD4-*bcl*6^{floxed} mice or their control littermates received tamoxifen (1 mg/day intraperitoneally for 5 consecutive days; Sigma Aldrich) **(See Fig. S3, S4)**.

With the exception of confocal microscopy experiments, for which C57BL6/J mice were adoptively transferred with 10^6 OT-1 CD45.1^{+} cells, naive mice received 10^4 purified OT-1 CD45.1^{+} cells retroorbitally. This number of transferred cells has been shown to prevent endogenous T cell responses to the SIINFEKL OVA epitope (Badovinac et al., 2007; Wirth et al., 2009). Two days later, mice were infected intravenously with 2.10^{4} CFU of a recombinant strain of *Listeria monocytogenes* expressing the 134–387 fragment of OVA protein as well as an erythromycin resistance gene (rLm-OVA was kindly provided by Prof. Hao Shen, Pennsylvania School of Medicine, USA) (Shen et al., 1995). For bacterial challenge at the memory stage, mice received 2.10^{5} CFU of rLm-OVA. Infection was carried out in a BSL2-level animal facility (Centre d'Exploration Fonctionnelle, UPMC, Paris).

Cell sorting and adoptive transfer experiments

For CXCR5^{+} and CXCR5^{-} OT-1 cell sorting, CD8^{+} T cells were enriched from splenocytes by using magnetic beads (CD8α MicroBeads, Miltenyi Biotec) on day 3.5 after rLm-OVA infection. Enriched CD8^{+} T cells were then stained with anti-CD45.2-V450, anti-CD4-V500, anti-CD45.1-PE-CF594, anti-CD3-Alexa700, anti-CXCR5-APC (all from BD Biosciences), anti-CD8-PE-Vio770 and anti-CD19-FITC (Miltenyi Biotec). Doublets were excluded on the basis of forward scatter (FSC) and side scatter (SSC) analysis (**See Fig. S1b**). CXCR5^{+} and CXCR5^{-} OT-1 cells were sorted in the CD19^{-} CD3^{+} CD4^{-} CD8^{+} CD45.2^{+} CD45.1^{+} cell gate.
To obtain Tfh and Tfh-depleted CD4-T cells, CD4 T cells were enriched from splenocytes by using magnetic beads (CD4 MicroBeads, Miltenyi Biotec) on day 3.5 after rLm-OVA infection, before staining with anti-CD8-V450, anti-CD4-V500, anti-CD3-Alexa700, anti-CXCR5-APC (all from BD Biosciences), anti-PD-1-FITC and anti-CD19-PE (Miltenyi Biotec). Highly purified CD4^{+} T cells were sorted from the CD19^{-} CD3^{+} CD8^{-} CD4^{+} cell gate. Tfh-depleted CD4^{+} T cells were defined as CD19^{-} CD3^{+} CD8^{-} CD4^{+} PD-1^{-} CXCR5^{-} cells and Tfh cells were defined as CD19^{-} CD3^{+} CD8^{-} CD4^{+} CXCR5^{+} PD-1^{hi} cells, as previously described (Crotty, 2011; Vinuesa et al., 2016). Cell sorting was performed with a FACS Aria III cell sorter (BD Biosciences) in a BSL2 laboratory.

Highly purified cells were then washed and resuspended in HBSS 1X. The following numbers of purified cells were injected retro-orbitally in a final volume of 100 µl: 10^4 OT-1 cells; 7.10^4 to 10^5 Tfh cells; 9.10^5 total $CD4^+$ T cells or Tfh-depleted $CD4^+$ T cells. In some experiments, Tfh and OT-1 cells were incubated for 1 hour at 37°C in the presence of 10 µg/ml of neutralizing anti-CD40L (clone MR1, BD Biosciences) or neutralizing anti-IL-21R (clone 4A9, BioXCell), respectively.

Flow cytometry

The following antibodies were used: anti-CXCR5-APC, anti-CCR7-BV650, anti-CD45.1-PE-CF594, anti-CD127-BV786, anti-CD3-Alexa 700, anti-KLRG1-BV421, anti-CD19-BV510, anti-IL21R-PE, CD40-PE-Cy7 and CD154-PE, anti-Bcl6-BV421 (BD Biosciences), anti-Granzyme-B-PE-Cy5 (eBioscience), anti-PD-1-FITC, anti-CD4- APC-Vio770, anti-CD8-PerCP-Vio700, anti-CD45.2-PE and anti-CD62L-FITC (Miltenyi Biotec). For Granzyme B staining we used the Cytofix/Cytoperm kit (BD Biosciences). For Bcl6 intracellular staining, we used the Foxp3 Staining Set (eBioscience). In some experiments, absolute cell counts were determined by means of TruCount tubes (BD Biosciences). Cells were acquired with a BD LSR Fortessa cytometer (BD Biosciences) and data were analyzed with FlowJo software. All mean fluorescence intensities (MFI) indicated in the manuscript correspond to geometric means.

Confocal microscopy

Sorted Tfh were stained with 5 µM CFSE (Sigma-Aldrich), then injected together with $CXCR5^+$ or $CXCR5^-$ OT-1 cells. In experiments designed to study interactions of OT-I with endogenous Tfh (**See Supp. Fig. 2e-f**), or effect of CXCL13 neutralization (**See Fig. 3d**), no Tfh were sorted and $CXCR5^+$ or $CXCR5^-$ OT-1 cells were injected alone. After 16 h, the mice were sacrificed and the spleens removed. Spleens were fixed in 4% paraformaldehyde, washed, incubated in sucrose solution, frozen in OCT (Sakura), then cryosectioned. Sections 14 µm thick were allowed to adhere to gelatin-coated glass slides (Gelatin, Sigma-Aldrich). The slides were then frozen at -80°C before staining.

The following antibodies were used (**in Fig. 3a-c and Supp. Fig. 2a**): for OT-1 detection, mouse anti-mouse-CD45.1 followed by goat anti-mouse-Cyanin3; for B cells, rat anti-mouse CD45R (clone B-220) followed by goat anti-rat-Alexa Fluor 647; for T cells, biotinylated anti-mouse CD3e followed by Streptavidin-Atto 425. Tfh were visualized by means of CFSE staining. All primary antibodies were from eBioscience and all secondary reagents were from Jackson Immunoresearch.

In Fig. 3d and Supp. Fig. 2e-f, the following antibodies were used: for OT-1 detection, mouse anti-mouse-CD45.1 followed by goat anti-mouse-Cyanin3; for B cells, rat anti-mouse CD45R (clone B-220) followed by goat anti-rat-Alexa Fluor 647; for T cells, biotinylated anti-mouse CD4 (BD Biosciences) followed by Streptavidin-Atto 425. For PD-1, rabbit anti-mouse PD-1 followed by goat anti-rabbit IgG-Alexa Fluor 488 (Both from Abcam). Slides were mounted with Vectashield (Vector Laboratories) and imaged with a confocal microscope (Leica SP5 with 20× or 63× lenses, NA 1.40). Images were analyzed with LSM Browser software (Zeiss).

Statistical analysis

Data were analyzed with GraphPad Prism. The Kruskal-Wallis and Mann-Whitney tests were used for unpaired data, and the Friedman and Wilcoxon tests for paired data.

SUPPLEMENTAL REFERENCES

Aghajani, K., Keerthivasan, S., Yu, Y., and Gounari, F. (2012). Generation of CD4CreER(T^2) transgenic mice to study development of peripheral CD4-T-cells. Genes. N. Y. N 2000 *50*, 908–913.

Badovinac, V.P., Haring, J.S., and Harty, J.T. (2007). Initial T cell receptor transgenic cell precursor frequency dictates critical aspects of the CD8(+) T cell response to infection. Immunity *26*, 827–841.

Buch, T., Heppner, F.L., Tertilt, C., Heinen, T.J.A.J., Kremer, M., Wunderlich, F.T., Jung, S., and Waisman, A. (2005). A Cre-inducible diphtheria toxin receptor mediates cell lineage ablation after toxin administration. Nat. Methods *2*, 419–426.

Crotty, S. (2011). Follicular Helper CD4 T Cells (TFH). Annu. Rev. Immunol. *29*, 621–663.

Hollister, K., Kusam, S., Wu, H., Clegg, N., Mondal, A., Sawant, D.V., and Dent, A.L. (2013). Insights into the role of Bcl6 in follicular Th cells using a new conditional mutant mouse model. J. Immunol. Baltim. Md 1950 *191*, 3705–3711.

Lee, P.P., Fitzpatrick, D.R., Beard, C., Jessup, H.K., Lehar, S., Makar, K.W., Pérez-Melgosa, M., Sweetser, M.T., Schlissel, M.S., Nguyen, S., et al. (2001). A critical role for Dnmt1 and DNA methylation in T cell development, function, and survival. Immunity *15*, 763–774.

Rickert, R.C., Roes, J., and Rajewsky, K. (1997). B lymphocyte-specific, Cre-mediated mutagenesis in mice. Nucleic Acids Res. *25*, 1317–1318.

Shen, H., Slifka, M.K., Matloubian, M., Jensen, E.R., Ahmed, R., and Miller, J.F. (1995). Recombinant Listeria monocytogenes as a live vaccine vehicle for the induction of protective anti-viral cell-mediated immunity. Proc. Natl. Acad. Sci. U. S. A. *92*, 3987–3991.

Vinuesa, C.G., Linterman, M.A., Yu, D., and MacLennan, I.C.M. (2016). Follicular Helper T Cells. Annu. Rev. Immunol. *34*, 335–368.

Wirth, T.C., Pham, N.-L.L., Harty, J.T., and Badovinac, V.P. (2009). High initial frequency of TCR-transgenic CD8 T cells alters inflammation and pathogen clearance without affecting memory T cell function. Mol. Immunol. *47*, 71–78.
